# Endoplasmic reticulum stress-mediated induction of SESTRIN 2 potentiates cell survival

**DOI:** 10.18632/oncotarget.7601

**Published:** 2016-02-22

**Authors:** Svetlana Saveljeva, Patricia Cleary, Katarzyna Mnich, Abiodun Ayo, Karolina Pakos-Zebrucka, John B Patterson, Susan E. Logue, Afshin Samali

**Affiliations:** ^1^ Apoptosis Research Centre, NUI Galway, Ireland; ^2^ School of Natural Sciences, NUI Galway, Ireland; ^3^ MannKind Corporation, Valencia, California, USA

**Keywords:** SESTRIN 2, ER stress, UPR, cell death, autophagy

## Abstract

Upregulation of SESTRIN 2 (SESN2) has been reported in response to diverse cellular stresses. In this study we demonstrate SESTRIN 2 induction following endoplasmic reticulum (ER) stress. ER stress-induced increases in SESTRIN 2 expression were dependent on both PERK and IRE1/XBP1 arms of the unfolded protein response (UPR). SESTRIN 2 induction, post ER stress, was responsible for mTORC1 inactivation and contributed to autophagy induction. Conversely, knockdown of SESTRIN 2 prolonged mTORC1 signaling, repressed autophagy and increased ER stress-induced cell death. Unexpectedly, the increase in ER stress-induced cell death was not linked to autophagy inhibition. Analysis of UPR pathways identified prolonged eIF2α, ATF4 and CHOP signaling in SESTRIN 2 knockdown cells following ER stress. SESTRIN 2 regulation enables UPR derived signals to indirectly control mTORC1 activity shutting down protein translation thus preventing further exacerbation of ER stress.

## INTRODUCTION

The mTORC1 complex is an important molecular sensor linking the cellular environment to key processes including proliferation and autophagy [1, 2]. Careful regulation of mTORC1 signaling is required to maintain cellular homeostasis especially following exposure to stress. Suppression of mTORC1 activity simultaneously reduces protein translation while stimulating pro-survival autophagy. SESTRIN 2 (SESN2), a member of the highly conserved stress-inducible Sestrin family, has been linked to mTORC1 regulation via AMP-activated protein kinase (AMPK/PRKA) [3]. SESTRIN 2 interaction with AMPK both activates and tethers AMPK to TSC2 leading to AMPK-mediated phosphorylation of TSC2 and subsequent inhibition of mTORC1 [3].

Stress-mediated induction of SESTRIN 2 has been predominantly associated with P53 signaling [4]. However, stress stimuli such as hypoxia, have been demonstrated to induce SESTRIN 2 via P53-independent means [4]. Recently endoplasmic reticulum (ER) stress has been linked to SESTRIN 2 regulation [5, 6]. ER stress can be induced by various physiological or chemical insults and is characterised by an accumulation of unfolded proteins within the lumen of the ER. Three ER transmembrane receptors IRE1 (ERN1), PERK (EIF2AK3) and ATF6 monitor the health of the ER and upon activation of ER stress act in concert to mediate the unfolded protein response (UPR). The UPR is an integrated signal transduction pathway, which aims to re-establish ER homeostasis through various adaptive mechanisms [7]. IRE1, PERK and ATF6 promote adaptation to protein-folding stress through the transcriptional upregulation of key ER chaperones, enhanced ER-associated degradation (ERAD) and inhibition of general protein translation. However, when ER stress is persistent, the UPR machinery can activate cell death pathways [8, 9]. Recent studies have identified ER stress as a mechanism through which SESTRIN 2 expression can be regulated. The proteasome inhibitor, Bortezomib (Btz), and HIV protease inhibitor, Nelfinavir, have both been demonstrated to induce SESTRIN 2 expression as a consequence of ER stress [6]. Likewise, Park and colleagues reported ER stress-mediated regulation of SESTRIN 2 in hepatocytes treated with palmitic acid [5]. How SESTRIN 2 expression is induced by ER stress is not fully understood although the PERK arm of the UPR appears to be required. Downstream targets of PERK, ATF4 and c/EBPβ (CEBPB), have been reported to regulate ER stress-induced SESTRIN 2 expression [5, 6].

In this study we demonstrate that induction of ER stress following treatment with pharmacological agents or chemotherapeutics such as Methotrexate (Mtx) lead to a P53-independent increase in SESTRIN 2. Our data indicates both IRE1/XBP1 and PERK branches of the UPR contribute to ER stress-induced SESTRIN 2 expression. Knockdown of *SESTRIN 2* reduced stress driven autophagy and enhanced cell death. Intriguingly, our results suggest that enhanced stress-induced cell death observed in *SESTRIN 2* knockdown cells is due to an exacerbation of ER stress rather than a decrease in autophagy.

## RESULTS

### ER stress induces SESTRIN 2 independent of P53

**Figure 1:**
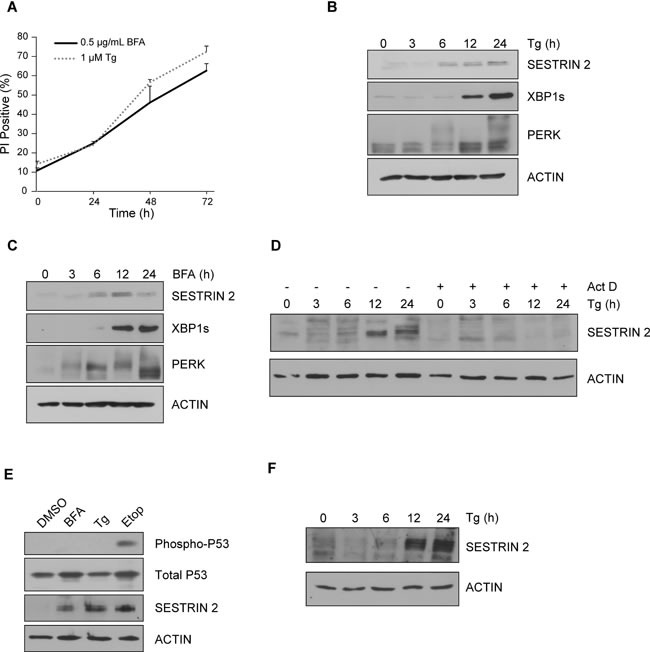
Induction of ER stress leads to upregulation of SESTRIN 2 expression independent of P53 MCF7 cells were treated with **A.** 0.5 μg/ml of BFA or 1 μM of Tg for the indicated time and viability assessed by propidium iodide (PI) uptake. Error bars represent the mean ± SD. MCF7 cells were treated with 0.5 μg/ml of Tg **B.** or 1 μM of BFA **C.** for the indicated time and lysates imunoblotted for SESTRIN 2, PERK, XBP1s. ACTIN was used as a loading control. **D.** MCF7 cells were treated with 1 μM of Tg alone or in combination with Act D (1 μg/ml) for the indicated time and lysates immunoblotted for SESTRIN 2 and ACTIN. **E.** MCF7 cells were treated with 0.5 μg/ml BFA, 1 μM Tg or 50 μM Etoposide (Etop) for 24 h after which lysates were immunoblotted for SESTRIN 2, Ser15 p-P53 and total P53. **F.** HCC1806 cells were treated with 1 μM Tg for the indicated time and cell lysates were then immunoblotted for SESTRIN 2 and ACTIN. Results are representative of at least 3 independent experiments.

Treatment of MCF7 cells with Thapsigargin (Tg) and Brefeldin A (BFA), two classical inducers of ER stress, was optimized to select doses which would permit examination of pro-survival and pro-death signaling over time (0.5 μg/ml BFA and 1 μM Tg) (Figure 1A). Tg and BFA, induced robust expression of SESTRIN 2 and activated the UPR as demonstrated by splicing of XBP1 and PERK phosphorylation (as determined by PERK upshift) (Figure 1B-1C). Similarly, SESTRIN 2 expression was induced by exposure to the ER stress inducer Dithiothreitol (DTT) (Supplemental Figure 1E). ER stress driven increases in SESTRIN 2 were transcriptionally mediated. Treatment with Tg increased *SESTRIN 2* mRNA levels (Supplemental Figure 1A) while, addition of Actinomycin D (Act D) prevented Tg-mediated SESTRIN 2 induction (Figure 1D). SESTRIN 2 regulation has been demonstrated to occur via P53 dependent and independent mechanisms [3, 5, 6]. Phosphorylation of P53 (Ser15), while readily detectable in cells treated with Etoposide (Etop), was not observed following either Tg or BFA treatment although a clear increase in the levels of SESTRIN 2 was evident (Figure 1E). Likewise, HCT116 *P53^+/+^* and *P53^−/−^* cells displayed Tg-induced SESTRIN 2 up-regulation, irrespective of P53 status. While, Etop-induced SESTRIN 2 expression was only observed in *P53^+/+^* cells (Supplemental Figure 1B-1C). Increased SESTRIN 2 expression was also detected in cells lacking wild-type P53 (HCC1806, K562) post Tg treatment (Figure 1F, Supplemental Figure 1D). Collectively, these data demonstrate the ability of ER stress to induce SESTRIN 2 expression in a P53 independent manner.

### UPR mediators contribute to ER stress-mediated induction of SESTRIN 2

**Figure 2:**
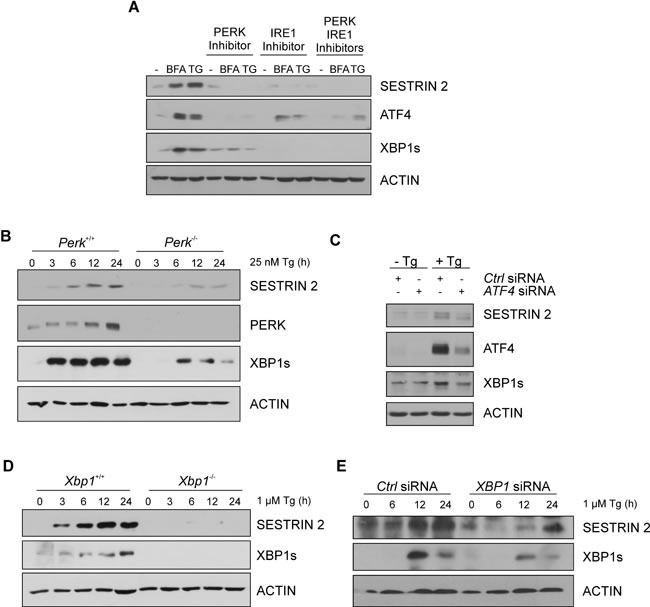
UPR pathways contribute to ER stress-induced enhancement of SESTRIN 2 expression **A.** MCF7 cells were treated with 0.5 μg/mL of BFA or 1 μM of Tg for 24 h alone or in combination with 300 nM PERK inhibitor, 10 μM IRE1 inhibitor or both. Levels of SESTRIN 2, ATF4, XBP1s and ACTIN were analysed by immunoblotting. **B.** *Perk*^+/+^ and *Perk*^−/−^ MEFs were treated with 25 nM Tg for the indicated time and lysates immunoblotted for SESTRIN2, PERK, XBP1s and ACTIN. **C.** MCF7 cells transfected with *ATF4* or Ctrl siRNA were treated with 1 μM Tg for 24 h and lysates immunoblotted for SESTRIN 2, XBP1s and ACTIN. **D.** *Xbp1*^+/+^ and *Xbp1*^−/−^ MEFs were treated with 1 μM of Tg for the indicated time and lysates immunoblotted for SESTRIN 2, XBP1s and ACTIN. **E.** MCF7 cells transfected with *XBP1* or Ctrl siRNA were treated with 1 μM Tg for the indicated time and lysates immunoblotted for SESTRIN 2, XBP1s and ACTIN. Results are representative of at least 3 independent experiments.

To understand how ER stress leads to an increase in SESTRIN 2 expression, we determined the contribution of each signaling arm of the UPR. siRNA knockdown of *ATF6*, while successful, did not inhibit Tg-induced increase in SESTRIN 2 (Supplemental Figure 2A). Addition of a PERK inhibitor alone, IRE1 inhibitor alone or a combination of both reduced Tg and BFA-mediated induction of SESTRIN 2 (Figure 2A). The functionality of each inhibitor was verified by examining their effect on downstream targets. Treatment with the PERK inhibitor, clearly attenuated ER stress-induced expression of ATF4, while inclusion of the IRE1 inhibitor blocked splicing of XBP1 (Figure 2A). To verify the role of PERK and determine if IRE1-mediated SESTRIN 2 induction is a result of IRE1/XBP1 signaling we examined SESTRIN 2 protein levels following ER stress in *Xbp1^−/−^* and *Perk^−/−^* mouse embryonic fibroblasts (MEF) cells. PERK-mediated translational inhibition is essential for cell survival following ER stress. Consequently, knockout of *Perk* renders cells exquisitely sensitive to ER stress-induced death [10]. For this reason *Perk^+/+^* and *Perk^−/−^* MEF cells were treated with a lower dose (25nM) of Tg as described previously [11]. *Perk^−/−^* MEF cells displayed lower SESTRIN 2 induction upon ER stress than their *Perk^+/+^* counterparts (Figure 2B). In agreement with previous studies, targeting the downstream PERK target *ATF4* also reduced Tg-mediated induction of SESTRIN 2 expression (Figure 2B-2C). Likewise, *Xbp1^−/−^* MEFs and MCF7 cells transfected with *XBP1* siRNA displayed a significant reduction in Tg-induced SESTRIN 2 induction underscoring a previously un-described role for XBP1 in SESTRIN 2 regulation (Figure 2D-2E).

### SESTRIN 2 knockdown modulates ER stress-induced autophagy and cell death responses

**Figure 3:**
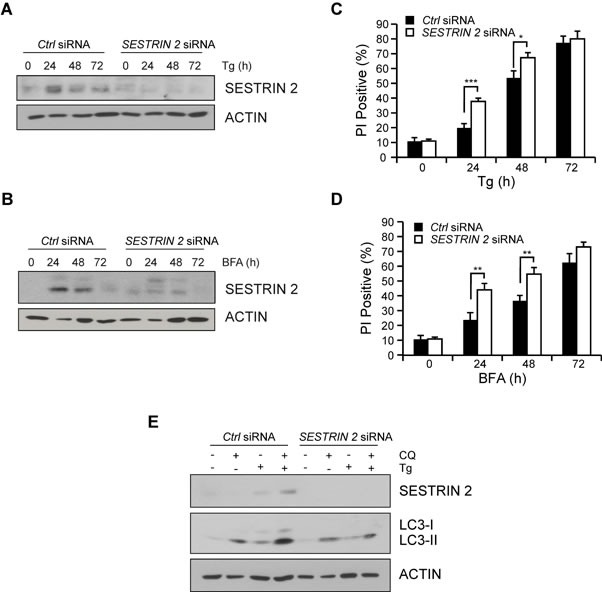
SESTRIN 2 knockdown inhibits ER stress-induced autophagy and enhances ER stress-induced cell death MCF7 cells transfected with *SESTRIN 2* or Ctrl siRNA were treated with 1 μM Tg (**A**, **C**) or 0.5 μg/ml BFA (**B**, **D**) for the indicated time after which lysates immunoblotted for SESTRIN 2 and ACTIN (**A**, **B**) and cell viability determined by PI uptake (**C**, **D**). **E.** Autophagic flux was evaluated in *SESTRIN 2* and Ctrl siRNA transfected MCF7 cells treated with 1 μM Tg for 24 h in the presence or absence of 20 μM chloroquine (CQ) and cell lysates immunoblotted for SESTRIN 2, LC3-I/II and ACTIN. Representative image of 3 independent experiments is shown.

To determine the relevance of SESTRIN 2 induction, MCF7 cells were transfected with siRNA against *SESTRIN 2* and subjected to ER stress. Knockdown was confirmed post Tg or BFA treatment by Western blotting (Figure 3A-3B). The outcome of *SESTRIN 2* knockdown on ER stress-induced cell death was determined by PI uptake. Cells transfected with *SESTRIN 2* siRNA displayed significantly higher ER stress-induced cell death compared to their control counterparts (Figure 3C-3D). This was especially evident at the earlier time-points, suggesting a role for SESTRIN 2 in the initial protective UPR response. Autophagy, an important pro-survival process, is induced following exposure to a range of stresses including ER stress [12]. Previous work has linked SESTRIN 2 to autophagy induction and cellular survival following genotoxic stress but little is known about its contribution during other types of stress. MTOR dephosphorylation and enhanced LC3-I (MAP1LC3) to LC3-II conversion was evident in cells treated with BFA or Tg suggesting autophagy induction (Supplemental Figure 3A-3B). Examination of GFP-LC3 distribution identified a punctate pattern indicative of autophagy induction upon ER stress (Supplemental Figure 3C). Addition of the autophagy inhibitor Spautin-1 (Spa-1) lowered the number of cells displaying punctate GFP-LC3 distribution following BFA treatment (Supplemental Figure 3C). To determine if SESTRIN 2 contributed to ER stress-induced autophagy, autophagic flux was examined in control and *SESTRIN 2* siRNA transfected MCF7 cells. Enhanced LC3-I to II conversion after co-treatment with chloroquine (CQ) was clearly evident in Tg-treated control siRNA cells. However, in *SESTRIN 2* siRNA-transfected cells significantly less LC3-I to II conversion was detected in cells treated with both Tg and CQ (Figure 3E). Likewise, *SESTRIN 2* knockdown inhibited autophagy (as determined by puncta formation) in BFA treated GFP-LC3 MCF7 (Supplemental Figure 3D).

### The chemotherapeutic Methotrexate induces ER stress and SESTRIN 2 expression via PERK and IRE1 signals

**Figure 4:**
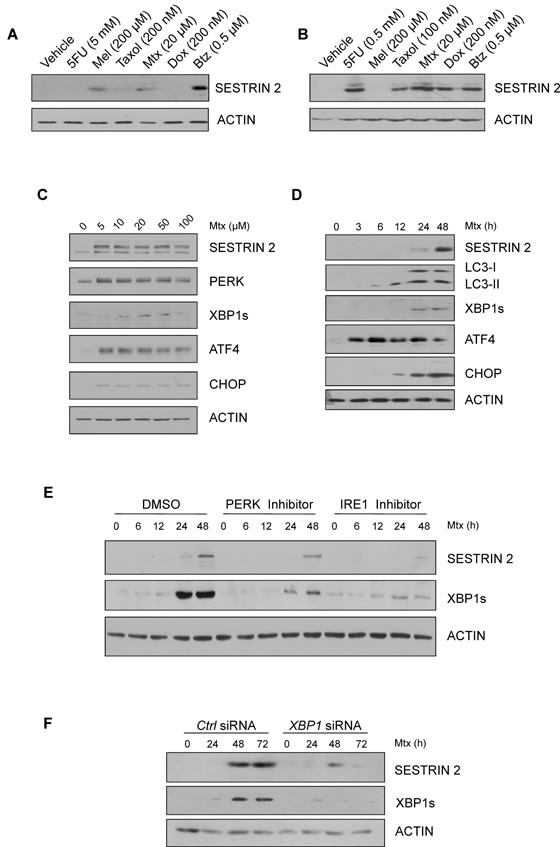
Chemotherapeutics, Methotrexate and Bortezomib, induce ER stress and SESTRIN 2 expression via UPR-mediated signals **A.-B.** HCC1806 (A) and MCF7 (B) cells were treated with 5 mM (A) 0.5 mM (B) 5-fluorouracil (5FU), 200 μM melphalan (Mel), 200 nM (A) 100 nM (B) Taxol, 20 μM Methotrexate (Mtx), 200 nM Doxorubicin, 0.5 μM Bortezomib (Btz) for 24 h and lysates immunoblotted for SESTRIN 2 and ACTIN. **C.** HCC1806 cells were treated with the indicated concentrations of Mtx for 48 h and lysates immunoblotted for SESTRIN 2, PERK, XBP1s, ATF4, CHOP and ACTIN. **D.** HCC1806 cells were treated with 20 μM Mtx for the indicated time and lysates immunoblotted for SESTRIN 2, LC3-I/II, XBP1s, ATF4, CHOP and ACTIN. **E.** HCC1806 cells were treated with 20 μM Mtx for the indicated time alone or in combination with 300 nM PERK or 10 μM IRE1 inhibitor and lysates were immunoblotted for SESTRIN 2, XBP1s and ACTIN. A representative image of 3 independent experiments is shown. **F.** Ctrl and *XBP1* siRNA-transfected HCC1806 cells were treated with 20 μM Mtx for the indicated time and lysates immunoblotted for SESTRIN 2, XBP1s and ACTIN.

Since our results indicated SESTRIN 2 expression is enhanced by ER stress we sought to determine if commonly used chemotherapeutics also induced SESTRIN 2 expression. Treatment of MCF7 and HCC1806 (triple negative *P53^−/−^* breast cancer cell line) cells with a range of chemotherapeutics detected elevated SESTRIN 2 expression following treatment with the anti-folate drug Mtx and the proteosomal inhibitor Btz (Figure 4A-4B). While treatment with Btz clearly induced ER stress (Supplemental Figure 4A) as has previously been reported [13], the induction of ER stress responses by Mtx has not yet been documented. RT-PCR confirmed Mtx, similar to Tg treatment, induced transcriptional upregulation of *SESTRIN 2* (Supplemental Figure 1F). Following treatment with Mtx, activation of both the PERK and IRE1 arms of the UPR was observed in MCF7 and HCC1806 cells confirming Mtx treatment, in addition to enhancing SESTRIN 2 expression, also triggered ER stress (Figure 4C-4D).

To determine the relevance of the UPR to Mtx-mediated SESTRIN 2 induction HCC1806 cells were pre-treated with PERK or IRE1 inhibitors prior to Mtx treatment and SESTRIN 2 expression was examined. Addition of either inhibitor reduced SESTRIN 2 expression, with the greatest effect observed in cells pre-treated with IRE1 inhibitor (Figure 4E). Knockdown of *XBP1* in HCC1806 cells showed a similar pattern, with reduced Mtx-mediated SESTRIN 2 induction in *XBP1* siRNA *versus* control siRNA-transfected cells (Figure 4F). Likewise in Btz-treated HCC1806 cells knockdown of *XBP1* reduced Btz driven expression of SESTRIN 2 (Supplemental Figure 4B). Collectively, our data suggest a model where treatment with chemotherapeutics such as Mtx and Btz induce ER stress, which in turn, via PERK and IRE1/XBP1 signals, drives SESTRIN 2 expression

### *SESTRIN 2* knockdown enhances Mtx-induced death in P53^−/−^ HCC1806 cells

**Figure 5:**
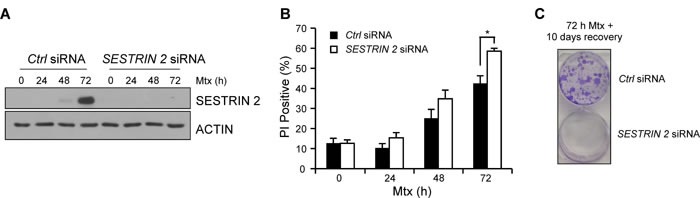
Knockdown of SESTRIN 2 enhances Methotrexate-induced death *Ctrl* and *SESTRIN 2* siRNAs transfected HCC1806 cells were treated 20 μM Mtx for the indicated time. **A.** Lysates were immunoblotted with SESTRIN 2 and ACTIN **B.** cell viability was determined by PI uptake. Mean of three independent experiments is shown ± SD. Statistical analysis was determined using t-Test. **C.** After transfection with Ctrl or *SESTRIN 2* siRNA HCC1806 cells were treated with 20 μM Mtx for 72 h. Mtx was removed and the colonies were left for 10 days before crystal violet staining. Representative image of two independent experiments is shown.

Since our earlier results, using specific ER stress-inducing agents, indicated knockdown of *SESTRIN 2* expression enhanced ER stress-induced cell death, we examined the outcome of *SESTRIN 2* knockdown on Mtx-induced death in HCC1806 cells. Examination of SESTRIN 2 expression following Mtx treatment confirmed knockdown over the 72 h time-course (Figure 5A). Similar to Tg and BFA, reduction of SESTRIN 2 expression via siRNA increased cell death in response to Mtx (Figure 5B). Owing to the lack of P53, HCC1806 cells are relatively resistant to Mtx-induced death. To examine the long term outcome of *SESTRIN 2* knockdown, HCC1806 cells were transfected with *SESTRIN 2* siRNA, followed by Mtx treatment for 72 h. After drug removal cells were left for an additional 10 days and colony formation was evaluated by crystal violet staining. While colonies were abundant in cells transfected with the control siRNA, the same was not true for cells transfected with the *SESTRIN 2* siRNA, where few if any colonies were visible (Figure 5C). This data indicates that *SESTRIN 2* knockdown enhances Mtx-induced death in HCC1806 cells in both the short and long term.

### *SESTRIN 2* knockdown reduces Mtx-induced autophagy independently of its pro-survival function

**Figure 6:**
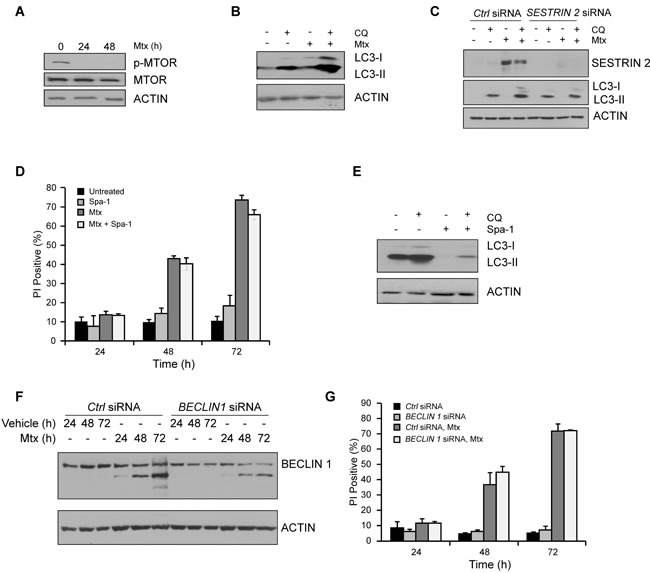
Impairment in autophagy does not account for enhanced Mtx-induced death observed in *SESTRIN 2* knockdown cells **A.** HCC1806 cells were treated with 20 μM Mtx for the indicated time after which lysates were immunoblotted for phospho-MTOR, total MTOR and ACTIN. **B.** Autophagic flux was evaluated in HCC1806 cells treated with 20 μM CQ alone, 20 μM Mtx alone, or a combination of 20 μM CQ and 20 μM Mtx for 48 h and cell lysates immunoblotted for SESTRIN 2, LC3-I/II and ACTIN. **C.** *Ctrl* and *SESTRIN 2* siRNAs transfected HCC1806 cells were treated with 20 μM Mtx ± 20 μM CQ for 48 h. Lysates were immunoblotted for SESTRIN 2, LC3-I/II and ACTIN. **D.** HCC1806 cells were treated with 20 μM Mtx alone, 10 μM Spautin-1 (Spa-1) alone or a combination of Mtx and Spa-1 for up to 72 h after which cell viability was assessed by PI uptake. **E.** HCC1806 cells were treated with 20 μM CQ alone, 10 μM Spa-1 alone or a combination of CQ and Spa-1 for 24 h and lysates immunoblotted for LC3-I/II and ACTIN. **F.**-**G.** *Ctrl* and *BECLIN1* siRNA transfected HCC1806 cells were treated with 20 μM Mtx for the indicated time. (F) Lysates were immunoblotted for SESTRIN 2 and ACTIN and (G) Cell viability assessed at each time-point by PI uptake.

To understand how *SESTRIN 2* knockdown enhanced Mtx-induced cell death we examined the contribution of autophagy. Following Mtx treatment, both MTOR dephosphorylation and enhanced LC3-I to II conversion was evident in HCC1806 cells (Figure 6A-6B). Knockdown of *SESTRIN 2*, while enhancing Mtx-induced cell death (Figure 5A-5B), also decreased Mtx-induced autophagy (Figure 6C). Likewise, SESTRIN 2 induction in Btz-treated HCC1806 cells was associated with enhanced autophagy (Supplemental Figure 4C). Similarly to Mtx-treated cells knockdown of *SESTRIN 2* both inhibited Btz-induced autophagy and enhanced cell death (Supplemental Figure 4D-4E). To determine if the reduced autophagy observed in Mtx-treated *SESTRIN 2* knockdown cells directly impacted upon cell death, an inhibitor of autophagy, Spa-1, was employed. Pre-treatment of cells with Spa-1, while clearly reducing autophagy, did not increase Mtx-induced cell death (Figure 6D-6E). In addition, knockdown of *BECLIN1* (*BECN1*) failed to sensitize cells to Mtx-induced death (Figure 6F-6G). Collectively, these observations suggest inhibition of autophagy is unlikely to contribute to the enhanced death observed in Mtx-treated HCC1806 cells following *SESTRIN 2* knockdown.

### SESTRIN 2 controls ER homeostasis by regulating MTOR activation in response to Mtx

**Figure 7:**
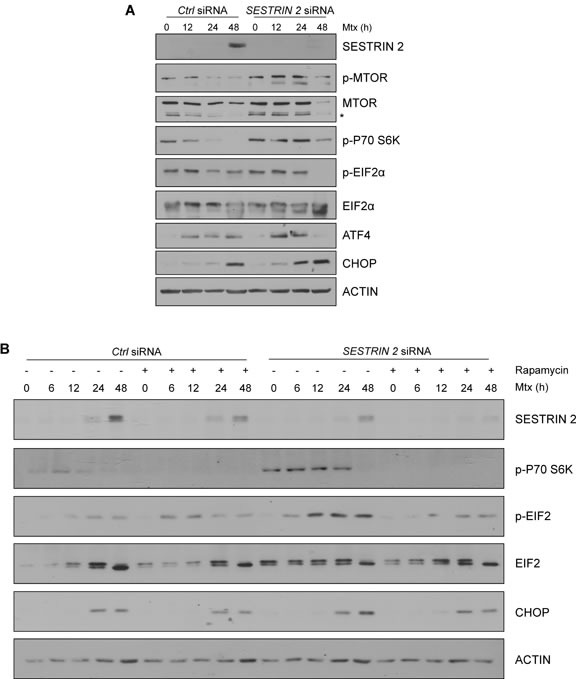
Knockdown of *SESTRIN 2* delays Mtx-induced dephosphorylation of MTOR and potentiates ER stress *Ctrl* and *SESTRIN 2* siRNAs transfected HCC1806 cells were treated with 20 μM Mtx for the indicated time. **A.** Lysates were immunoblotted with SESTRIN 2, phospho-MTOR, total MTOR, phospho-P70 S6K, phospho-EIF2α, total EIF2α, ATF4, CHOP and ACTIN. **B.** Ctrl and *SESTRIN 2* siRNAs transfected HCC1806 cells were treated 20 μM Mtx ± 100 nM Rapamycin for the indicated time. Lysates were immunoblotted for SESTRIN 2, phospho-P70 S6K, phospho-EIF2α, total EIF2α, CHOP and ACTIN. A representative image of 3 independent experiments is shown.* Denotes non-specific band.

Recently it has been reported that SESTRIN 2 expression is critical for inhibition of protein translation during ER stress [5]. It was demonstrated that *SESTRIN 2* deficiency resulted in persistent protein synthesis under ER stress conditions resulting in enhanced ER stress-induced death [5]. To examine if this was also the case in our model, levels of MTOR phosphorylation were examined in HCC1806 cells following Mtx treatment. Clear inactivation, as determined by MTOR dephosphorylation, was observed in Mtx and Btz-treated HCC1806 cells 24 h post treatment (Figure 6A, Supplemental Figure 4F). Knockdown of *SESTRIN 2* abrogated MTOR dephosphorylation and prolonged P70 S6K (RPS6KB1) phosphorylation following Mtx treatment (Figure 7A). Lower SESTRIN 2 expression has been previously linked to an increase in UPR signals and in particular enhanced activation of the PERK arm of the UPR [5]. To determine whether *SESTRIN 2* knockdown potentiates PERK-mediated signaling following Mtx-induced ER stress we examined ATF4, CHOP (DDIT3) and EIF2α (EIF2S1) phosphorylation in Mtx-treated control *versus SESTRIN 2* siRNA transfected cells. Prolonged activation of PERK signaling was observed in Mtx-treated cells transfected with *SESTRIN 2* siRNA, suggesting *SESTRIN 2* knockdown potentiates Mtx-induced ER stress in HCC1806 cells (Figure 7A-7B). Similar results were also observed with Tg demonstrating this is due to increased ER stress *per se* and not specifically Mtx treatment (Supplemental Figure 5A). To ascertain if the enhancement in ER stress-induced markers observed in *SESTRIN 2* siRNA cells was a direct result of sustained MTOR activity we utilised an inhibitor of MTOR, rapamycin. Treatment of *SESTRIN 2* siRNA HCC1806 cells with rapamycin reduced both Mtx-induced mTORC1 activity, as determined by phospho-P70 S6K, and also reduced *SESTRIN 2* knockdown-mediated enhancement of PERK signaling (Figure 7B).

## DISCUSSION

Microarray studies within our laboratory had previously identified *SESTRIN 2* as an ER stress regulated gene [14]. In this study, we demonstrate ER stress regulation of SESTRIN 2 expression independent of P53. How SESTRIN 2 is induced following ER stress is not entirely understood. Park and colleagues reported PERK-mediated SESTRIN 2 induction via c/EBPβ, while Bruning *et al* found SESTRIN 2 induction to be ATF4 dependent [5, 6]. In our system ER stress-mediated upregulation of SESTRIN 2 was dependent on both IRE1 and PERK signals. Reduced SESTRIN 2 expression was evident in both *Perk* and *Xbp1* deficient MEF cells upon exposure to ER stress. Knockdown of *XBP1* in MCF7 and HCC1806 cells attenuated Tg and Mtx-mediated induction of SESTRIN 2. This data suggests enhancement of SESTRIN 2 expression upon ER stress requires both IRE1/XBP1 and PERK-mediated signals. Such a co-operative induction is unsurprising as ER stress sensors rarely function in a singular distinct way but rather in a highly co-ordinated manner. For example, we observed decreased XBP1 splicing in both *Perk*^−/−^ MEFs and cells treated with PERK inhibitor following ER stress, indicating PERK signaling is required for robust activation of the XBP1. Indeed reduced *Xbp1* mRNA expression has been previously reported in *Perk*^−/−^ MEFs following tunicamycin treatment [15]. Based on these observations it seems likely that reducing/blocking one branch of the UPR can have far reaching effects on other branches of this signaling pathway.

To examine the therapeutic relevance of targeting SESTRIN 2 we examined the ability of a panel of commonly used chemotherapeutics to induce SESTRIN 2 expression in MCF7 cells and the P53 null triple negative breast cancer cell line HCC1806. Treatment with Mtx and or Btz potently induced SESTRIN 2 expression in both cell lines. As the triple negative HCC1806 cell line is devoid of P53 we chose to use this cell line for all further experiments. Examination of both Mtx and Btz-treated cells revealed, that in addition to inducing SESTRIN 2, both drugs triggered ER stress. It has been well established that Btz is a potent inducer of ER stress [13]. However, a role for Mtx as a novel inducer of ER stress has not previously been characterised. Again, as observed with Tg and BFA treatment of MCF7 cells, knockdown of *SESTRIN 2* expression decreased Mtx-induced autophagy and enhanced cell death in HCC1806 cells. Since autophagy is predominantly considered a pro-survival process we reasoned enhanced Mtx-induced death in *SESTRIN 2* knockdown cells is a direct consequence of lowered autophagy. However, studies using the autophagy inhibitor Spautin-1 or knockdown of *BECLIN1* argued against this. Following siRNA knockdown of *SESTRIN 2* Mtx-induced dephosphorylation of MTOR was reduced. Associated with this we observed an enhancement of PERK-mediated signaling indicating a potentiation of ER stress. Inhibition of MTOR signaling in Mtx-treated *SESTRIN 2* siRNA cells, by addition of rapamycin, lowered PERK driven signals as determined by attenuated EIF2α phosphorylation and CHOP induction. Unexpectedly, addition of rapamycin to control and *SESTRIN 2* siRNA cells treated with Mtx also lowered SESTRIN 2 expression (Figure 7B). This reduction may be a consequence of reduced protein translation by rapamycin-mediated inhibition of mTORC1.

**Figure 8:**
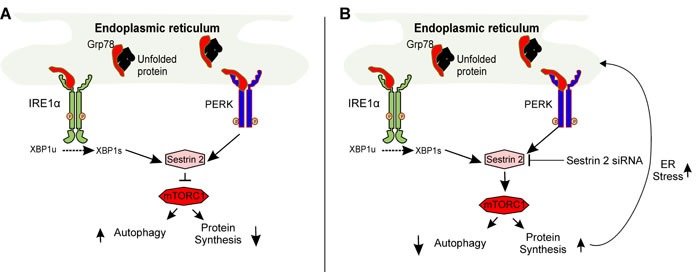
Schematic representation of SESTRIN 2-mediated signaling during ER stress **A.** Accumulation of unfolded proteins within the ER lumen (ER stress) triggers activation of IRE1 and PERK upregulating SESTRIN 2 expression. SESTRIN 2 via interaction with AMPK and TSC2 inhibits MTORC1 activity decreasing protein translation and increasing autophagy. **B.** Knockdown of *SESTRIN 2* prevents MTORC1 inhibition resulting in sustained protein translation and autophagy inhibition. This in turn, places further stress on the ER exacerbating ER stress and leading to enhanced cell death.

In agreement with our findings, exacerbation of ER stress as a consequence of *SESTRIN 2* deficiency has recently been reported. Park and colleagues demonstrated sustained mTORC1 activity leading to elevated ATF4 and CHOP expression in *Sestrin 2* deficient cells upon induction of ER stress [5]. Increased ATF4 and CHOP expression has been demonstrated to antagonize translational inhibition by inducing genes involved in protein synthesis triggering oxidative stress and ultimately cell death [16]. Enhancement of SESTRIN 2 expression, by ER stress, blocks protein translation via mTORC1 inactivation promoting cellular adaption and restoration of ER homeostasis. This supports the hypothesis that SESTRIN 2 expression protects breast cancer cells from Mtx-induced cell death by means of inhibiting protein synthesis through inhibition of mTORC1 rather than autophagy induction (Figure 8).

This study highlights the pro-survival role of SESTRIN 2 and demonstrates targeting of SESTRIN 2 in breast cancer cells represents a mechanism to potentiate ER stress-induced cell death. SESTRIN 2 induction has been reported in other cancer models including skin cancer where it was also linked cell survival [17]. These results, together with the results of our study, highlight the importance SESTRIN 2 induction during cell stress.

## MATERIALS AND METHODS

### Cell culture and treatments

MCF7 (ATCC), HCC1806 (ATCC), K562 (ATCC) cells were cultured in either Dulbecco's modified Eagle's medium (DMEM) (Sigma #D6429) (MCF-7) or Roswel Park Memorial Institute medium (RPMI 1640) (Sigma #R0883) (HCC1806, K562) supplemented with 10% heat inactivated fetal bovine serum (HI FBS, Sigma #7524), 100 U/ml penicillin/100 mg/ml streptomycin (Sigma #P0781) and 2 mM L-glutamine (Sigma #G7513). HCT116 colon cancer cells were cultured in McCOYs 5A medium modified (Sigma #M9309) supplemented with 10% HI FBS, 100 U/ml penicillin/100 mg/ml streptomycin. *Xbp1^−/−^* and *Xbp1^+/+^* control MEFs (Dr. Laurie Glimcher, Harvard Medical School, USA) were maintained in DMEM medium supplemented with 10% HI FBS, 2 mM L-glutamine, 1 mM sodium pyruvate (Sigma #S8636), non-essential amino acid solution (Sigma #M7145). *Perk^+/+^* and *Perk^−/−^* MEFs (Prof. David Ron Institute of Metabolic Science, University of Cambridge, UK) were maintained in DMEM medium supplemented with 10% HI FBS, 2 mM L-glutamine, 1 mM sodium pyruvate, non-essential amino acid solution, 55 μM β-mercaptoethanol (βME), 1% penicillin/streptomycin. All cells were cultured at 37°C, 5% CO_2_ in a humidified incubator. Cells were seeded at an appropriate number 24 h prior to treatment. 5-Fluorouracil (Sigma #F6627), Paclitaxel (Sigma #T7402), Doxorubicin (LC laboratories #D-4000), Melphalan (Sigma #M2011), Bortezomib (Selleck Chemicals #PS-341), Methotrexate (Sigma #M9929), Brefeldin A (Sigma #B7651), Thapsigargin (Sigma #T9033), Etoposide (Sigma #E1383), Rapamycin (LC-laboratories #R-5000), IRE1 inhibitor, MKC-8866, (Mannkind Corporation), Spautin-1 (Sigma #SML0440) and PERK inhibitor, GSKG797800 (Toronto Research Chemicals).

### Flow cytometry

Cells were collected by trypsinization and subsequent centrifugation, resuspended in ice-cold PBS containing 0.7 μg/ml of propidium iodide (Sigma #P4170) and analysed using FACSCalibur flow cytometer (Becton Dickinson).

### Western blotting

Cells were washed once in ice-cold PBS and lysed in whole cell lysis buffer (4% SDS, 120 mM Tris HCl, 10% glycerol, 100 mM DTT and dash of bromphenol blue) and boiled at 95°C for 5 min. Equal amounts of protein samples were run on an SDS polyacrylamide gel. The proteins were transferred onto nitrocellulose membrane and blocked with 5% milk in PBS-0.1% Tween. For detection of protein expression the following antibodies were used: ACTIN (Sigma #A5060), LC3 (Sigma #L7543), SESTRIN 2 (Sigma #WH0083667M3), XBP1 (Abcam #619502), ATF4 (CST #11815), CHOP (CST #2895), MTOR (CST #2983) p-MTOR (CST #2974), PERK (CST #3192), p-P70 S6K (CST #9234). All the secondary antibodies were purchased from Jackson and the signal was visualized using Western Blotting Luminol Reagent (SantaCruz sc-2048).

### Gene silencing and overexpression

MCF7 and HCC1806 cells were transfected using Lipofectamine 2000 (Life Technologies, #11668019) according to the manufacturer's protocol. siRNAs (ON-TARGET plus smart pool) were obtained from Dharmacon *SESTRIN 2* (L-019134-02-0005); *XBP1* (L-009552-00-0005); *ATF6* (L-009917-00-0005); *ATF4* (L-005125-00-0005); *BECLIN1* (L-010552-00-0005); Non-coding siRNA (D-001810-01-20). Media was changed 4 h post-transfection and cells were left to recover for 24 h. After the recovery, cells were reseeded for the experiment that was performed 36 h post-transfection.

GFP-LC3 plasmid was transiently transfected into MCF7 cells according to manufacturers protocol using Lipofectamine 2000 (Life Technologies, #11668019). 24 h post transfection cells were treated as indicated and GFP-LC3 positive punctate *versus* diffuse cells (3 fields of 100 cells) were counted.

### RNA extraction and cDNA synthesis

Total RNA was isolated from cells using TRI Reagent (Invitrogen) according to the manufactures protocol. To synthesize cDNA, 2 μg of RNA was subjected to DNase treatment and incubated for 15 min at room temperature. The RNA was then reverse transcribed into cDNA using Superscript III first strand RT-PCR system and random hexamers (Invitrogen). The cDNA product was subjected to PCR using the forward primer 5′-CAGAGGGCACAGGAAAGAAG-3′ and the reverse primer 5′-GAACTAGGATTCGGGCAACA-3′ for the detection of human *SESTRIN 2*. *GAPDH* was used as an endogenous control using the forward primer 5′-ACCACAGTCCATGCCATC-3′ and reverse 5′-TCCACCACCTGTTGCTG-3′.

### Clonogenic assay

HCC1806 cells were transfected with *SESTRIN 2* and non-coding control siRNAs as described above. 36 h later they were treated with 20 μM of Mtx for 72 h. After the treatment, media was removed and cells were left to recover for 10 days. Colonies were stained with 2% crystal violet in 20% methanol.

### Statistical analysis

Cell death and data is expressed as mean ± SD for three independent experiments. Real Time data is expressed as mean ± SEM for three independent experiments. Differences between the treatment groups were assessed using Graphpad's Two-tailed unpaired student's t-tests. The values with ^*^p < 0.05 is considered statistically significant, **p < 0.01, ^***^p < 0.001.

## SUPPLEMENTARY MATERIAL FIGURES


