# Supplementary material for: Endoplasmic reticulum stress-mediated induction of SESTRIN 2 potentiates cell survival
Source: Oncotarget. 2016 Feb 22;7(11):12254–66. doi: 10.18632/oncotarget.7601 (PMC4914282; doi:10.18632/oncotarget.7601)
Supplement: Supplementary file 1 [file oncotarget-07-12254-s001.pdf]

# Endoplasmic reticulum stress-mediated induction of SESTRIN 2 potentiates cell survival

**Supplementary Material**

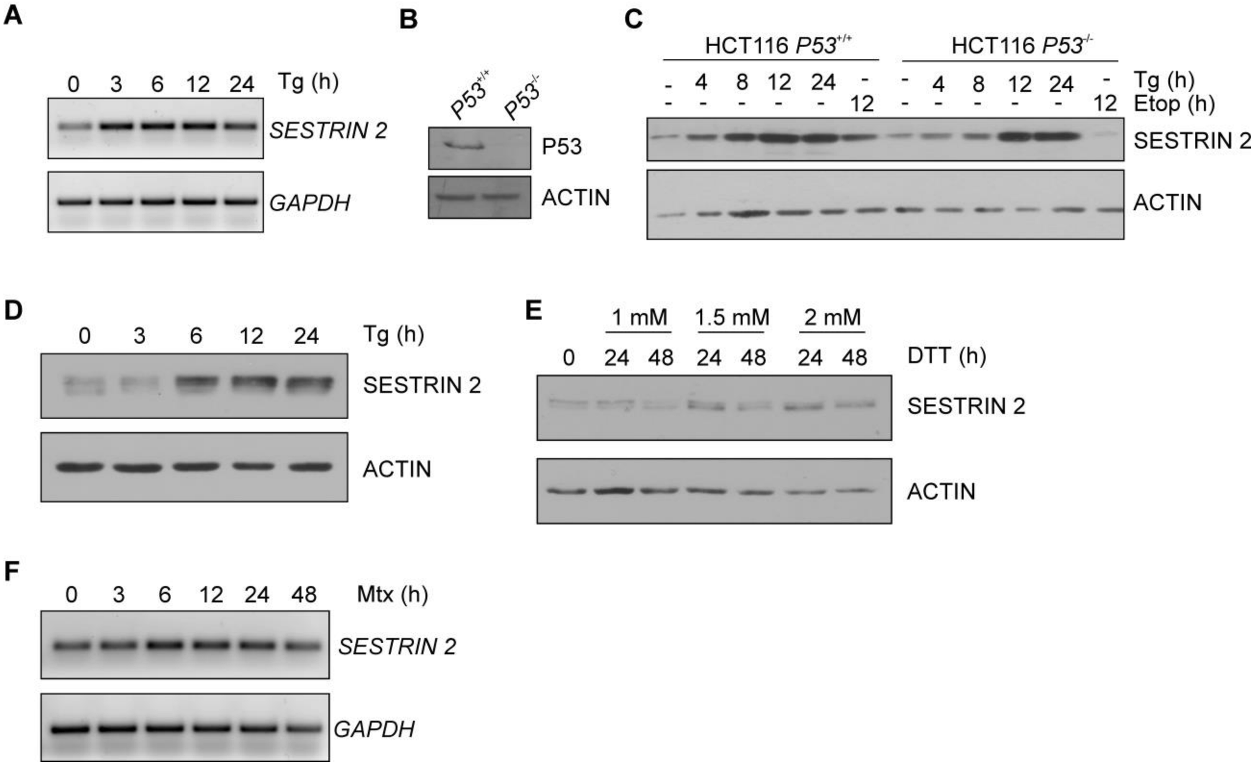


**Supplementary Figure 1. ER stress transcriptionally upregulates SESTRIN2 in a P53 independent manner.**

(**A**) HCC1806 cells were treated for the indicated time with 1 μM Tg and *SESTRIN 2* and *GAPDH* mRNA levels were examined by RT-PCR (**B**) Lysate from HCT116 *P53*$^{+/+}$ and *P53*$^{-/-}$ cells were immunoblotted for total P53 and ACTIN (**C**) HCT116 *P53*$^{+/+}$ and *P53*$^{-/-}$ cells were treated with 1 μM Tg for 4 - 24 h or 50 μM Etoposide (Etop) for 12 h and lysates immunoblotted for SESTRIN 2 and ACTIN**.** (**D**) K562 cells were treated with 1 μM Tg for the indicated time and cell lysates were then immunoblotted for SESTRIN 2 and ACTIN. (**E**) MCF7 cells were treated for the indicated time with 1, 1.5 and 2 mM

DTT. SESTRIN 2 and ACTIN expression was determined by immunoblotting (**F**) HCC1806 cells were treated for the indicated time with 20 µM Mtx and *SESTRIN 2* and *GAPDH* mRNA levels examined by RT-PCR. A representative image of 3 independent experiments is shown.

**A**

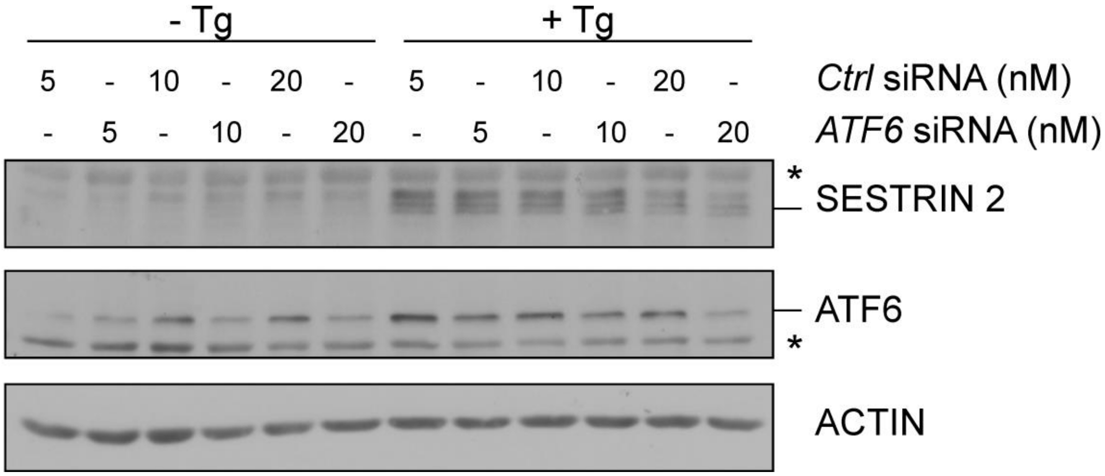


**Supplementary Figure 2. ATF6 does not contribute to ER stress-induced upregulation of SESTRIN 2.**

(**A**) *Ctrl* and *ATF6* siRNAs transfected MCF7 cells were treated ± 1 µM Tg for the 24 h and lysates immunoblotted for SESTRIN 2, ATF6 and ACTIN. * Denotes non-specific band.

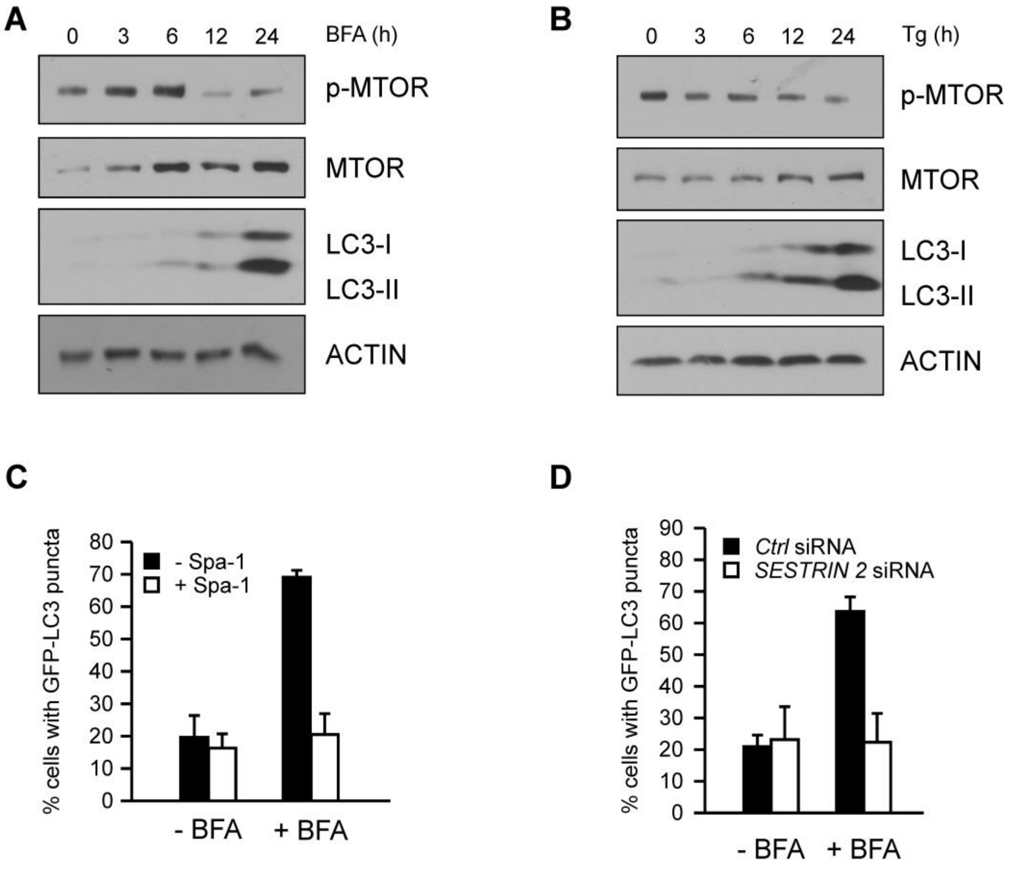


**Supplementary Figure 3. Induction of ER stress triggers MTOR dephosphorylation and autophagy in MCF7 cells.**

MCF7 cells were treated for the indicated time with 0.5 µg/ml BFA (**A**) or 1 µM Tg (**B**) cell lysates were then immunoblotted for phospho-MTOR, total MTOR, LC3-I/II and ACTIN. (C) MCF7 cells transiently transfected with GFP-LC3 were treated with 0.5 µg/ml BFA ± 10 µM Spa-1 and the percentage of GFP-LC3 positive punctate cells counted at 12 h. (D) GFP-LC3 MCF7 cells transfected with Ctrl and *SESTRIN 2* siRNAs transfected were treated ± 0.5 µg/ml BFA for 12 h and the percentage of GFP-LC3 expressing cells positive for punctate staining determined. Three fields of at least 100 cells/field were counted.

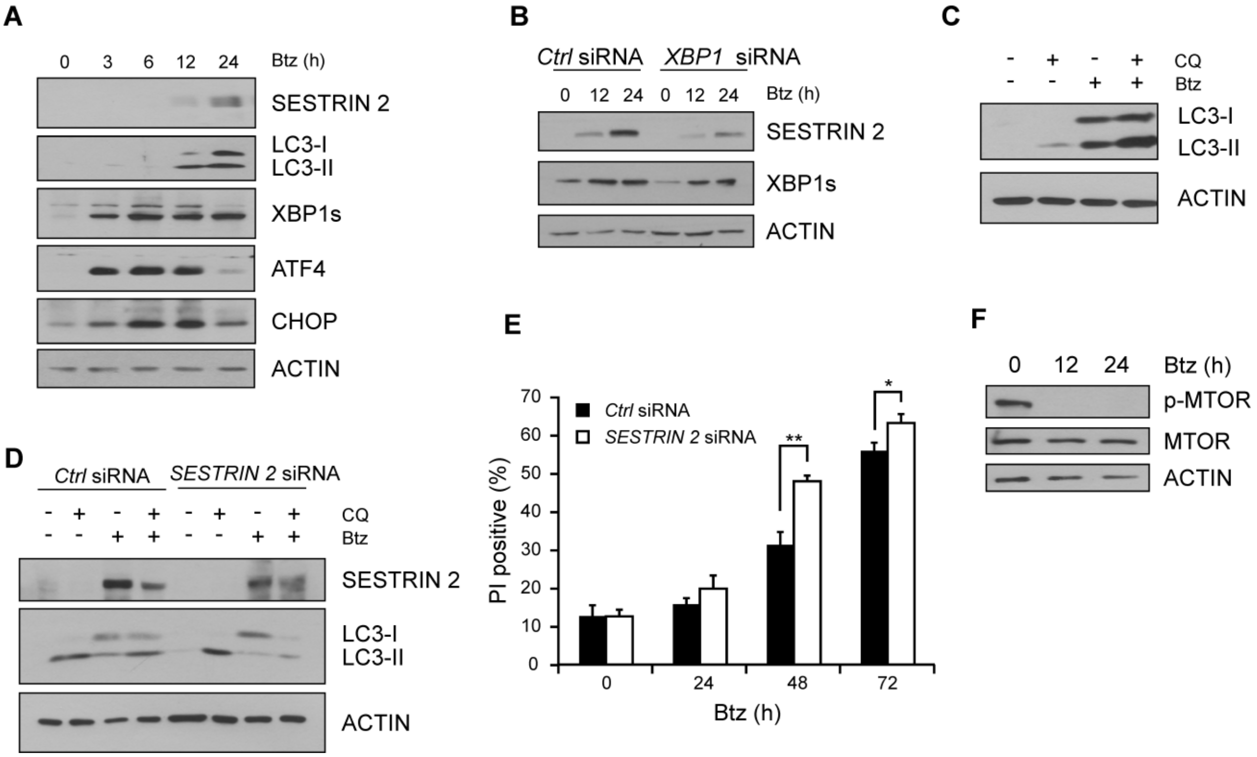


**Supplementary Figure 4. Bortezomib treatment triggers ER stress, MTOR dephosphorylation and autophagy in HCC1806 cells.**

(**A**) HCC1806 cells were treated with 0.5 µM of Btz for indicated time and lystes immunoblotted for SESTRIN 2, LC3-I/II, XBP1s, ATF4, CHOP and ACTIN. (**B**) *Ctrl* and *XBP1* siRNA transfected HCC1806 cells were treated 0.5 µM Btz for the indicated time and lysates immunoblotted for SESTRIN 2, XBP1s and ACTIN. (**C**) HCC1806 cells treated with 0.5 µM Btz for 24 h with or without 20 µM of CQ and lysates immunoblotted for LC3-I/II and ACTIN. (**D**) Ctrl and *SESTRIN 2* siRNAs transfected HCC1806 cells were treated 0.5 µM Btz for 24 h with or without 20 µM of CQ and lysates immunoblotted for SESTRIN 2, LC3-I/II and ACTIN (**E**) Ctrl and *SESTRIN 2* siRNAs transfected HCC1806 cells were treated 0.5 µM Btz for the indicated time and cell death assessed via PI uptake. Mean of three independent experiments is shown and

statistical analysis was determined by t-Test (**F**) HCC1806 cells were treated 0.5 µM Btz for the indicated time and lysates immunoblotted for phospho-MTOR, total MTOR and ACTIN. Results from 3 independent experiments were presented as a mean.

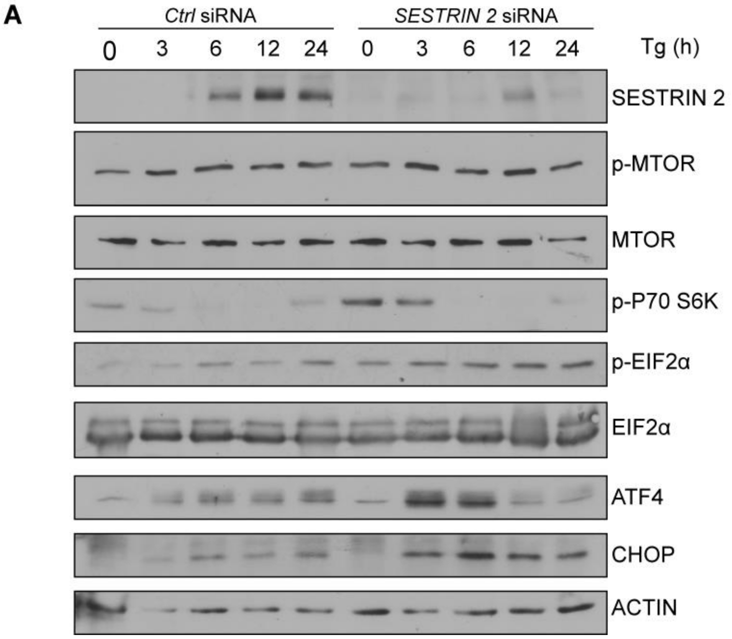


**Supplementary Figure 5. Knockdown of *SESTRIN 2* delays Tg-induced dephosphorylation of MTOR and potentiates ER stress**

(**A**) *Ctrl* and *SESTRIN 2* siRNAs transfected HCC1806 cells were treated 1 µM Tg for the indicated time. Lysates were immunoblotted with SESTRIN 2, phospho-MTOR, total MTOR, phospho-P70 S6K, phospho-EIF2α, total EIF2α, ATF4, CHOP and ACTIN.
